# Development and validation of a competing risk model for second primary pancreatic ductal adenocarcinoma: A population-based study

**DOI:** 10.3389/fsurg.2022.934148

**Published:** 2022-08-30

**Authors:** Lishan Song, Chaojie Xu, Tong Zhang, Shengyang Chen, Zhigang Shi, Shuiquan Hu, Bingbing Cheng, Hao Tong, Guangkun Wei, Xiaoyong Li

**Affiliations:** The Fifth Affiliated Hospital of Zhengzhou University, Zhengzhou University, Zhengzhou, China

**Keywords:** second primary malignancy, SEER database, competing regression analysis, nomogram risk, pancreatic ductal adenocarcinoma

## Abstract

**Background:**

With advances in early diagnosis and treatment, the number of cancer survivors continues to grow, and more and more cancer survivors face the threat of second primary cancer (SPM). Second primary pancreatic ductal adenocarcinoma (spPDAC) is an important subclass of SPM, but its prognostic characteristics are poorly understood.

**Methods:**

A total of 5,439 spPDAC samples and 67,262 primary pancreatic ductal adenocarcinoma (pPDAC) samples were extracted from the SEER database for this study. Survival differences between spPDAC and pPDAC samples were compared using Kaplan–Meier curves and log-rank tests. The Fine and Gray proportional subdistributed hazard method was used to analyze potential associations between clinical variables and pancreatic ductal adenocarcinoma-specific death (PDACSD) and death from other causes. After that, the clinical variables significantly related to PDACSD were screened out to construct a competing risk nomogram, which was used to evaluate the probability of the occurrence of PDACSD. The C-index was used to evaluate the discriminative ability of the model. The area under the curve (AUC) was used to verify the discrimination of the model. The calibration curve was used to verify the calibration of the model. Decision curve analysis (DCA) was used to validate the clinical utility of the model.

**Results:**

Compared with patients with spPDAC, the pPDAC sample had a better prognosis (*p* = 0.0017). Across all spPDAC samples, the three most common sites of first-present cancer were the prostate, breast, and digestive system. Age (*p* < 0.001), race (*p* = 0.006), interval (*p* = 0.016), location (*p* < 0.001), T stage (*p* = 0.003), M stage (*p* < 0.001), chemotherapy (*p* < 0.001), and radiotherapy (*p* = 0.006) were the clinical variables associated with PDACSD screened by multivariate competing risks analysis. The concordance index values for the training and validation sets were 0.665 (95% CI, 0.655, 0.675) and 0.666 (95% CI, 0.650, 0.682), respectively. AUC, calibration curve, and DCA indicated that the model we constructed had good discrimination, calibration, and clinical utility.

**Conclusions:**

In conclusion, we first analyzed the impact of previous cancer history on prognosis. We then constructed a competing risk model that can predict the probability of developing PDACSD in spPDAC. This model has good discriminative ability, calibration, and clinical practicability and has certain guiding value for clinical decision-making.

## Introduction

Second primary malignancy (SPM) refers to the reappearance of a new primary malignant tumor based on the original malignant tumor (1). The number of cancer survivors is also growing due to early diagnosis, advances in treatment technology, and an aging population (2). Some statistical agencies predict that in 2026, there will be 20 million cancer survivors (3). Cancer survivors represent approximately 3.5% of the general population in the United States, and approximately one in ten newly diagnosed cancers occurs in cancer survivors (4, 5). Statistics show that with an increase in the number of cancer survivors, the number of patients with SPM also has a steady upward trend (6, 7). SPM has emerged as a significant risk factor for cancer survivors. First primary cancers (FPCs) and their treatments may influence the biological progression, treatment, and prognosis of SPM (8–11). This has led many studies to exclude this particular group. However, the increasing number of SPM patients urgently needs more research to provide guidance for clinical decision-making.

Second primary pancreatic ductal adenocarcinoma (spPDAC) is an important component of SPM. As one of the most common cancers worldwide, pancreatic ductal adenocarcinoma (PDAC) is the seventh leading cause of cancer-related death (12, 13). As more and more cancer survivors are at risk from SPM, the development of PDAC to SPM is also more frequent (14, 15). A pooled analysis study of international multicenter cancer registries reported that spPDAC accounted for 6.9% of all PDAC diagnoses (15). A Korean study showed that the type of FPC can affect the probability and prognosis of spPDAC (16). In a cohort study based on 273,144 samples, an increased incidence of pancreatic cancer was found in a population of patients with previous colon cancer (17). Due to the characteristics of multiple primary cancers, a large number of studies have excluded this special group. Furthermore, because the occurrence of spPDAC cases is relatively rare and difficult to collect, there is currently a lack of research on the prognostic characteristics of spPDAC. There are few studies on spPDAC, and the risk factors associated with spPDAC remain unclear.

The aim of this study was to analyze the impact of previous cancer on the prognosis of spPDAC patients and to identify clinical and demographic factors associated with spPDAC survival. Based on the Fine and Gray proportional subdistributed hazard method, we attempted to create competing risk nomograms to predict half-year, 1-year, and 2-year pancreatic ductal adenocarcinoma-specific mortality for spPDAC.

## Materials and methods

### Data sources

The data used in this study were extracted from the Surveillance, Epidemiology, and End Results (SEER) database (https://seer.cancer.gov/). Using SEER*Stat (version 8.4.0) data extraction software, eligible samples from the 18 population-based registriy (2000–2018) datasets were downloaded (18). Based on submissions in November 2020 and released in April 2021, the dataset covers 18 regions, including San Francisco-Oakland SMSA, Connecticut, and Detroit (Metropolitan), and accounts for 27.8% of the total US population. For Group A, 16,392 samples with a history of cancer were extracted. The retrieval conditions are as follows: (1) the first tumor was malignant; (2) the second primary cancer was in the pancreas; and (3) the histological diagnosis was positive. Extract clinical variables of interest include gender, ethnicity, age and year at diagnosis, site of cancer occurrence, pathological type, marital status, location of spPDAC, TNM stage of spPDAC, treatment of spPDAC, FPC site, and FPC histology type. For Group B, 67,945 pPDAC samples were extracted. The search criteria are as follows: (1) age not less than 20 years old; (2) the time of diagnosis was between 2004 and 2015; (3) the topographic code located in the pancreas was selected (ICD-O-3: C25.0–C25.3, C25 .7–C25.9) with ICD-O-3 histology/behavior code 8140/3 (adenocarcinoma) or 8500/3 (invasive ductal adenocarcinoma); and (4) only one primary malignancy occurred. This study was exempt from institutional review board approval due to the public nature and deidentification of all data.

### Data processing

**Figure 1:**
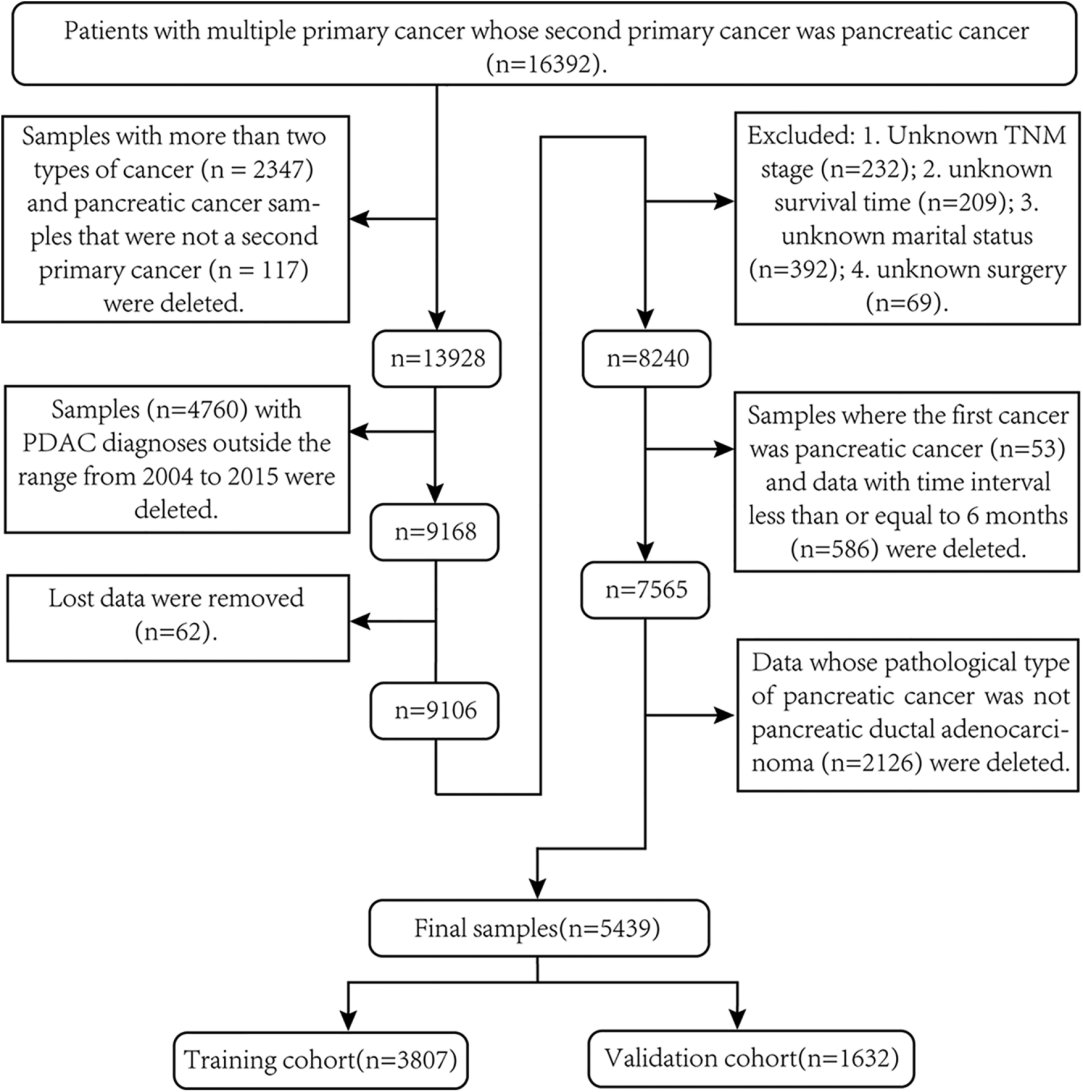
Screening process for second primary pancreatic ductal adenocarcinoma samples required for this study.

Of the 16,392 original samples in group A, 5,439 samples were finally screened for follow-up studies. The exclusion criteria are as follows: (1) delete samples with three or more primary tumors (*n* = 2,347); (2) delete samples with pancreatic cancer as the third and fourth primary cancers (*n* = 117); (3) samples (*n* = 4,760) whose spPDAC diagnosis time was not within the time range from 2004 to 2015 were deleted; (4) delete missing data (*n* = 62); (5) delete missing clinical variables (*n* = 866); (6) delete samples where the FPC was pancreatic cancer (*n* = 53) and samples (*n* = 586) where the interval between two cancers was less than or equal to 6 months; and (7) exclude patients whose pathological type of SPM is not pancreatic ductal adenocarcinoma (*n* = 2,126). To screen out samples that fit clinicopathological types, we first used the International Classification of Neoplastic Diseases to select topographic codes with primary sites located in the pancreas (ICD-O-3: C25.0–C25.3, C25.7–C25.9) (19). Second, samples with ICD-O-3 histology/behavior code 8140/3 (adenocarcinoma) or 8500/3 (invasive ductal adenocarcinoma) were selected (19–21). The detailed process of data screening is shown in Figure 1. After removing samples with unknown data from the 67,945 samples in group B (race unknown, *n* = 129; surgical status unknown, *n* = 441; survival time unknown, *n* = 113), the remaining 67,262 samples were used for follow-up studies.

### Statistical analysis

Numbers, percentage values, medians, quartiles, means, and variances were used to describe extreme baseline data. Survival differences between spPDAC and pPDAC samples were compared using KM survival curves and log-rank tests. All spPDAC samples were divided into a training set (*n* = 3,807) and a validation set (*n* = 1,632) according to the ratio of 7:3. The chi-square test was used to verify whether there were differences between categorical variables in the training and validation sets. Two independent sample *t*-tests were used to verify whether there was a difference in the interval between two primary cancers in the training and validation sets.

Causes of death were divided into pancreatic ductal adenocarcinoma-specific death (PDACSD) and death from other causes (DFOC). However, DFOC includes deaths from the first primary cancer. For the two events, PDACSD and DFOC, since one occurs, the other will not occur, so DFOC is an important competing event for PDACSD.

The Fine and Gray proportional subdistributed hazard method was used to analyze risk factors for PDACSD and DFOC. Using the risk factors of PDACSD, a competing risk model was constructed to predict the probability of PDACSD occurring in 6 months, 1 year, and 2 years. The effects of individual factors on the probability of occurrence of PDACSD and DFOC were analyzed by the univariate Fine–Gray test using the cumulative incidence function (CIF) (22, 23). The concordance index (C-index), the area under the curve (AUC), and the calibration curve were used to verify the accuracy and discrimination of the model. Decision curve analysis (DCA) (24) was used to analyze the benefit of patients after using the model.

All data analyses in this study were performed in R (version R-4.1.3). The “survival” and “survminer” packages were used for KM analysis and the log-rank test. The “chisq.test” package was used for chi-square tests. The “t.test” package was used for two independent sample *t*-tests. In the “cmprsk” package, the “crr()” function was used for the multivariate analysis of competing risk models, and the “cuminc()” function was used for the univariate Fine–Gray test. Packages “mstate,” “rms,” and “regplot” were used to draw competing risk nomograms. The “timeROC” and “survivalROC” packages were used to draw the area under the receiver operating characteristic curve. The “calPlot” package was used to draw calibration curves in competing risk models. The “Stdca” package is used to draw DCA (24). In all statistical tests in this paper, a two-sided *p* value less than 0.05 was considered statistically different.

### Ethical statement

The authors are accountable for all aspects of the work in ensuring that questions related to the accuracy or integrity of any part of the work are appropriately investigated and resolved. Institutional review board approval was waived for this study because the SEER database is a public anonymized database. All of the methods we used in this study were carried out in accordance with relevant guidelines and regulations.

## Results

### Baseline characteristics of the study population

After a series of screening, 5,439 spPDAC samples and 67,262 pPDAC samples were finally included in the study. As shown in Table 1, spPDAC and pPDAC samples differed significantly in terms of gender, age, race, and marital status. Compared with spPDAC, pPDAC samples were younger and had more females. In addition, the TNM stage was relatively high in pPDAC samples. The baseline characteristics of the spPDAC sample are shown in Table 2, and the median (interquartile range, IQR) values of time to diagnosis for FPC and spPDAC were 2005 (2003, 2008) and 2011 (2008, 2014), respectively. The median ages at diagnosis for FPC and spPDAC were 68 (61, 75) and 73 (66, 80) years, respectively. The mean ages at diagnosis of FPC and spPDAC were 67.45 (9.81) and 72.67 (9.64) years, respectively. The median (IQR) of the time interval between the diagnosis of two primary cancers was 55 (28, 89) months, and the mean (standard deviation, SD) was 62.61 (41.15) months. The median (IQR) from spPDAC diagnosis to endpoint, competing event, or end of the study was 5 (2, 13) months. More than half of the patients (61.37%) used chemotherapy after the diagnosis of spPDAC. Only a small number of patients underwent surgery (18.75%) and radiotherapy (17.26%).

**Table 1:** Demographic characteristics of patients.

	spPDAC (*n* = 5,439), *n* (%)	pPDAC (*n* = 67,262), *n* (%)	*p*
Sex, *n* (%)			<0.001
Female	2,212 (40.67)	32,662 (48.56)	
Male	3,227 (59.33)	34,600 (51.44)	
Age, year, *n* (%)			<0.001
<65	1,105 (20.31)	29,018 (43.14)	
≥65	4,334 (79.68)	38,244 (56.86)	
Race, *n* (%)			<0.001
White	4,476 (82.29)	53,440 (79.39)	
Black	643 (11.82)	8,455 (12.57)	
Other	320 (5.89)	5,367 (8.00)	
Marital status, *n* (%)			<0.001
Unmarried	2,065 (37.97)	30,017 (44.63)	
Married	3,374 (62.03)	37,245 (55.37)	
Site, *n* (%)			0.7347
PancreasHead	2,804 (51.55)	34,786 (51.72)	
PancreasBodyTail	1,354 (24.89)	16,934 (25.18)	
OthPancreas	1,281 (23.55)	15,542 (23.11)	
T stage, *n* (%)			<0.001
TX/1/2	2,437 (44.81)	28,063 (41.72)	
T3/4	3,002 (55.19)	39,199 (58.28)	
N stage, *n* (%)			<0.001
NX/0	3,702 (68.06)	43,882 (65.24)	
N1	1,737 (31.94)	23,380 (34.76)	
M stage, *n* (%)			<0.001
MX/0	2,849 (52.38)	32,531 (48.36)	
M1	2,590 (47.62)	34,731 (51.64)	
Surgery, *n* (%)			0.8882
Yes	1,020 (18.75)	12,562 (18.68)	
No	4,419 (81.25)	54,700 (81.32)	
Chemotherapy, *n* (%)			<0.001
Yes	2,794 (51.37)	36,896 (54.85)	
No	2,645 (48.63)	30,366 (45.15)	
Radiotherapy, *n* (%)			0.0151
Yes	939 (17.26)	12,507 (18.59)	
No	4,500 (82.74)	54,755 (81.41)	

TNM stage based on 6th edition staging of the American Joint Commission on Cancer.

**Table 2:** Overview of demographic and clinical factors in spPDAC patients.

At prior cancer diagnosis (*n* = 5,439)		At spPDAC diagnosis, (*n* = 5,439)	
Variables	Value	Variables	Value
Year of diagnosis		Year of diagnosis	
Median (IQR)	2005 (2003, 2008)	Median (IQR)	2011 (2008,2014)
Age, year		Age, year, *n*	
Mean (SD)	67.45 (9.81)	Mean (SD)	72.67 (9.64)
Median (IQR)	68 (61, 75)	Median (IQR)	73 (66, 80)
Sex, *n* (%)		Sex, *n* (%)	
Female	2,212 (40.67)	Female	2,212 (40.67)
Male	3,227 (59.33)	Male	3,227 (59.33)
Race, *n* (%)		Race, *n* (%)	
White	4,476 (82.29)	White	4,476 (82.29)
Black	643 (11.82)	Black	643 (11.82)
Other	320 (5.89)	Other	320 (5.89)
Marital status, *n* (%)		Marital status, *n* (%)	
Unmarried	1,702 (31.29)	Unmarried	2,065 (37.97)
Married	3,335 (61.32)	Married	3,374 (62.03)
Unknown	402 (7.39)	Unknown	∼
T stage, *n* (%)		Site, *n* (%)	
TX\1\2	2,689 (49.44)	PancreasHead	2,804 (51.55)
T3\4	533 (9.80)	PancreasBodyTail	1,354 (24.89)
Unknown	2,217 (40.76)	OthPancreas	1,281 (23.55)
N stage, *n* (%)		T stage, *n* (%)	
NX\0	3,194 (58.72)	TX\1\2	2,437 (44.81)
N1	28 (0.51)	T3\4	3,002 (55.19)
Unknown	2,217 (40.76)	N stage, *n* (%)	
M stage, *n* (%)		NX\0	3,702 (68.06)
MX\0	3,140 (57.73)	N1	1,737 (31.94)
M1	82 (1.51)	M stage, *n* (%)	
Unknow	2,217 (40.76)	MX\0	2,849 (52.38)
Surgery, *n* (%)		M1	2,590 (47.12)
Yes	3,901 (71.72)	Surgery, *n* (%)	
No	1,514 (27.84)	Yes	1,020 (18.75)
Unknow	24 (0.44)	No	4,419 (81.25)
Chemotherapy, *n* (%)		Chemotherapy, *n* (%)	
Yes	992 (18.24)	Yes	2,794 (51.37)
No	4,447 (81.76)	No	2,645 (48.63)
Radiotherapy, *n* (%)		Radiotherapy, *n* (%)	
Yes	1,774 (32.62)	Yes	939 (17.26)
No	3,665 (67.38)	No	4,500 (82.74)
Interval between diagnoses, months		Time from spPDAC diagnosis to death or end of study, months	
Mean (SD)	62.61 (41.15)	Mean (SD)	9.09 (10.23)
Median (IQR)	55 (28, 89)	Median (IQR)	5 (2, 13)

Data were *n* (%)unless otherwise specified. IQR, interquartile range; spPDAC, Second primary pancreatic ductal adenocarcinoma; SD, standard deviation; ∼, Not detectable.

**Figure 2:**
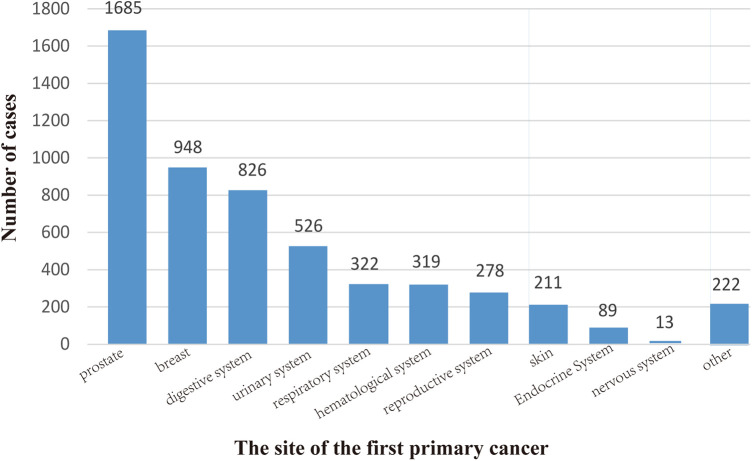
Location of the first primary cancer. We divided it into 11 sites, the most common of which is the prostate (1685), followed by the breast (948) and the digestive system (826). The locations of 5,439 cases are shown here.

As shown in Figure 2, the three sites with the most FPCs were the prostate (*n* = 1,685), breast (*n* = 948), and digestive system (*n* = 826).

### Influence of previous cancer history on prognosis

**Figure 3:**
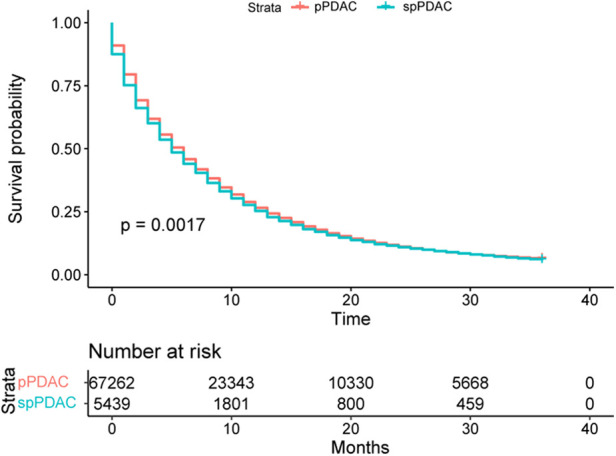
KM curve analysis of the difference in prognosis between spPDAC and pPDAC samples.

At the end of follow-up (time = 3 years), 5,127 patients in the spPDAC group had died, accounting for 94.26% of the total study sample. In the pPDAC subgroup, 63,050 samples died at the end of follow-up, accounting for 93.74% of the total sample. As shown, we plotted KM survival curves and validated them using the log-rank test (Figure 3). The results showed that patients without a history of cancer had a better prognosis (*p* = 0.0017).

### Cause of death analysis of spPDAC subgroups

**Figure 4:**
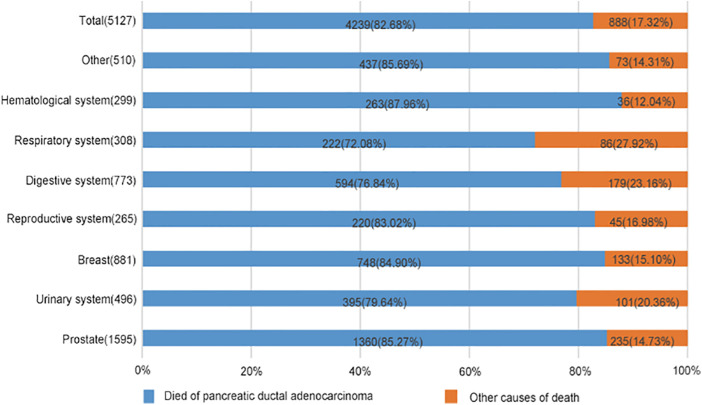
Percentage of spPDAC cancer-specific and other-cause-specific deaths, by location of first primary cancer. Compare the proportions of causes of death in this graph.

As shown in Figure 4, 4,239 patients died from spPDAC, leaving 888 patients from FPC or other causes. As can be seen from the figure, spPDAC was the leading cause of death in spPDAC patients regardless of the location of the FPC. Compared with other systems, the respiratory system, digestive system, and urinary system had lower PDACSD, accounting for 72.08%, 76.84%, and 79.64%, respectively. The proportion of PDACSD in other parts was more than 80%.

### Baseline characteristics of the training set and validation set

In a 7:3 ratio, the total study spPDAC sample (*n* = 5,439) was randomly divided into a training set (*n* = 3,807) and a validation set (*n* = 1,632). As shown in Table 3, gender, age, race, marital status, location of spPDAC, TNM stage, surgical treatment, chemotherapy, and the time interval between two primary cancers were not statistically different in the training set and validation set. The training set was used for the development and internal validation of the competing risk model. The validation set was used for external validation of the model. In the training cohort, there were more males, accounting for 59.50%, and the majority were elderly, accounting for 79.96%. More than half of the patients (51.12%) had spPDAC in the head of the pancreas. Most of the patients did not undergo surgery (81.46%) and radiotherapy (83.43%) after the diagnosis of spPDAC. About half of the patients (51.04%) used chemotherapy.

**Table 3:** Demographics of training and validation sets.

	Total (*n* = 5,439), *n* (%)	Training set (*n* = 3,807), *n* (%)	Validation set (*n* = 1,632), *n* (%)	*p*
Sex, *n* (%)				0.7053
Female	2,212 (40.67)	1,542 (40.50)	670 (41.05)	
Male	3,227 (59.33)	2,265 (59.50)	962 (58.95)	
Age, year, *n* (%)				0.4427
<65	1,105 (20.31)	763 (20.04)	342 (20.96)	
≥65	4,334 (79.68)	3,044 (79.96)	1,290 (79.04)	
Race, *n* (%)				0.9611
White	4,476 (82.29)	3,136 (82.37)	1,340 (82.11)	
Black	643 (11.82)	447 (11.74)	196 (12.01)	
Other	320 (5.89)	224 (5.88)	96 (5.88)	
Marital status, *n* (%)				0.1500
Unmarried	2,065 (37.97)	1,469 (38.59)	596 (36.52)	
Married	3,374 (62.03)	2,338 (61.41)	1,036 (63.48)	
Interval, month				0.9643
Mean (SD)	62.61 (41.15)	62.63 (41.06)	62.58 (41.36)	
Median (IQR)	55 (28, 89)	55 (28,88)	55 (28,89)	
Site, *n* (%)				0.0804
PancreasHead	2,804 (51.55)	1,946 (51.12)	858 (52.57)	
PancreasBodyTail	1,354 (24.89)	980 (25.74)	374 (22.92)	
OthPancreas	1,281 (23.55)	881 (23.14)	400 (24.51)	
T stage, *n* (%)				0.9889
TX/1/2	2,437 (44.81)	1,706 (44.81)	731 (44.79)	
T3/4	3,002 (55.19)	2,101 (55.19)	901 (55.21)	
N stage, *n* (%)				0.4390
NX/0	3,702 (68.06)	2,579 (67.74)	1,123 (68.81)	
N1	1,737 (31.94)	1,228 (32.26)	509 (31.19)	
M stage, *n* (%)				0.2327
MX/0	2,849 (52.38)	1,974 (51.85)	875 (53.62)	
M1	2,590 (47.62)	1,833 (48.15)	757 (46.38)	
Surgery, *n* (%)				0.5471
Yes	1,020 (18.75)	706 (18.54)	314 (19.24)	
No	4,419 (81.25)	3,101 (81.46)	1,318 (80.76)	
Chemotherapy, *n* (%)				0.4541
Yes	2,794 (51.37)	1,943 (51.04)	851 (52.14)	
No	2,645 (48.63)	1,864 (48.96)	781 (47.86)	
Radiotherapy, *n* (%)				0.0399
Yes	939 (17.26)	631 (16.57)	308 (18.87)	
No	4,500 (82.74)	3,176 (83.43)	1,324 (81.13)	

TNM stage based on 6th edition staging of American Joint Commission on Cancer.

### Competitive risk analysis

We divided the causes of death into PDACSD and DFOC and used the Fine and Gray proportional subdistributed hazard method to analyze the risk factors for death of patients (Table 4). Age (*p* < 0.001), race (*p* = 0.006), interval (*p* = 0.016), location (*p* < 0.001), T stage (*p* = 0.003), M stage (*p* < 0.001), chemotherapy (*p* < 0.001), and radiotherapy (*p* = 0.006) were risk factors for PDACSD. From Table 4, we can find that patients who were older at diagnosis [subdistribution hazard ratio (sdHR) 1.225 (95% CI, 1.121–1.338)] were more likely to develop PDACSD. The higher the clinical T (sdHR = 1.130, 95% CI, 1.043–1.224) and M (sdHR = 1.279, 95% CI, 1.172–1.397) stage, the higher the probability of PDACSD. Compared with no chemotherapy or radiotherapy, chemotherapy (sdHR = 0.733, 95% CI, 0.678–0.793) and radiotherapy (sdHR = 0.888, 95% CI, 0.810–0.973) could significantly reduce the incidence of PDACSD. Compared with White, black (sdHR = 0.818, 95% CI, 0.720–0.929) and other skin-colored races (sdHR = 0.888, 95% CI, 0.758–1.040) were less likely to develop PDACSD. The longer the interval between FPC diagnosis and spPDAC (sdHR = 1.001, 95% CI, 1.000–1.002), the higher the probability of PDACSD.

**Table 4:** Competing risk models for mortality from pancreatic ductal adenocarcinoma and death from other causes.

Characteristics	Death from spPDAC		Death from other causes	
	sdHR (95%CI)	*p*	sdHR (95%CI)	*p*
Sex		0.780		0.023
Female	Reference		Reference	
Male	0.994 (0.920–1.074)	0.880	1.192 (1.001–1.418)	0.048
Age		<0.001		0.010
<65	Reference		Reference	
≥65	1.225 (1.121–1.338)	<0.001	0.786 (0.646–0.955)	0.015
Race		0.006		0.004
White	Reference		Reference	
Black	0.818 (0.720–0.929)	0.002	1.660 (1.347–2.046)	<0.001
Other	0.888 (0.758–1.040)	0.140	1.085 (0.772–1.526)	0.640
Marital status		0.950		0.054
Unmarried	Reference		Reference	
Married	0.991 (0.913–1.075)	0.820	0.887 (0.747–1.055)	0.180
Interval	1.001 (1.000–1.002)	0.016	0.997 (0.995–0.999)	0.005
Site		<0.001		<0.001
PancreasHead	Reference		Reference	
PancreasBodyTail	1.006 (0.921–1.099)	0.890	1.023 (0.834–1.256)	0.830
OthPancreas	0.817 (0.738–0.905)	<0.001	1.526 (1.260–1.849)	<0.001
T stage		0.003		<0.001
TX/1/2	Reference		Reference	
T3/4	1.130 (1.043–1.224)	0.003	0.698 (0.587–0.830)	<0.001
N stage		0.280		0.660
NX/0	Reference		Reference	
N1	1.051 (0.968–1.141)	0.240	1.035 (0.860–1.246)	0.710
M stage		<0.001		0.760
MX/0	Reference		Reference	
M1	1.279 (1.172–1.397)	<0.001	0.981 (0.817–1.179)	0.840
Surgery		0.000		0.840
No	Reference		Reference	
Yes	0.518 (0.469–0.573)	0.000	1.010 (0.792–1.287)	0.940
Chemotherapy		<0.001		<0.001
No	Reference		Reference	
Yes	0.733 (0.678–0.793)	<0.001	0.593 (0.496–0.709)	<0.001
Radiotherapy		0.006		0.310
No	Reference		Reference	
Yes	0.888 (0.810–0.973)	0.011	1.101 (0.866–1.401)	0.430

sdHR, subdistribution hazard ratio; CI, confidence interval; TNM stage based on the 6th edition staging of the American Joint Commission on Cancer (AJCC); spPDAC, second primary pancreatic ductal adenocarcinoma.

Similarly, gender (*p* = 0.023), age (*p* = 0.010), race (*p* = 0.004), time interval (*p* < 0.001), specific location of spPDAC (*p* < 0.001), T stage (*p* < 0.001), and chemotherapy (*p* < 0.001) were associated with the occurrence of DFOC. DFOC includes not only deaths due to FPC but also other causes of death such as car accidents and cardiovascular disease.

**Figure 5:**
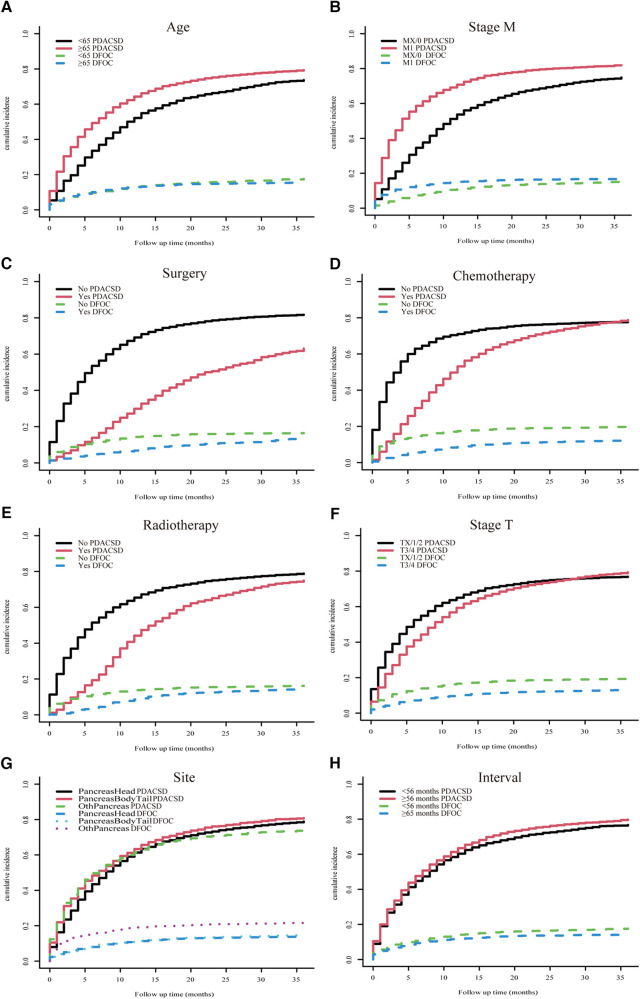
Univariate Fine–Gray test was used to analyze the cumulative incidence of pancreatic ductal adenocarcinoma-specific death and death from other causes. Age (**A**), M stage (**B**), surgery (**C**), chemotherapy (**D**), radiotherapy (**E**), T stage (**F**), location (**G**), and time interval (**H**).

The univariate Fine–Gray test showed that the cumulative probability of occurrence of PDACSD and DFOC showed significant differences when the values of individual clinical variables were different (Figure 5).

### Development and validation of a pancreatic ductal adenocarcinoma-specific mortality nomogram

**Figure 6:**
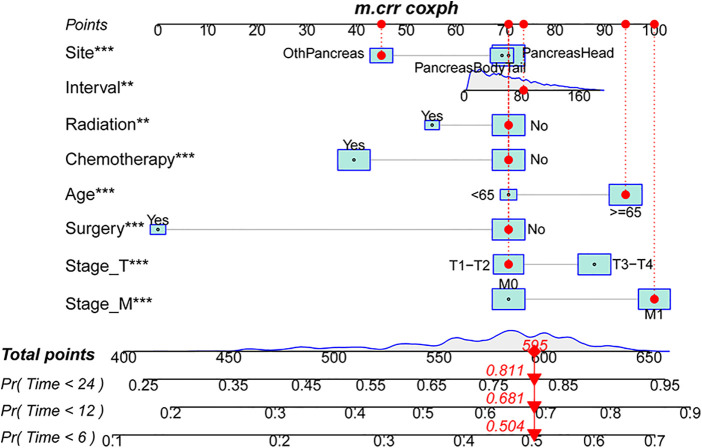
Nomogram for predicting 6-month, 1-year, and 2-year pancreatic ductal adenocarcinoma-specific mortality in patients with second primary pancreatic ductal adenocarcinoma.

**Figure 7:**
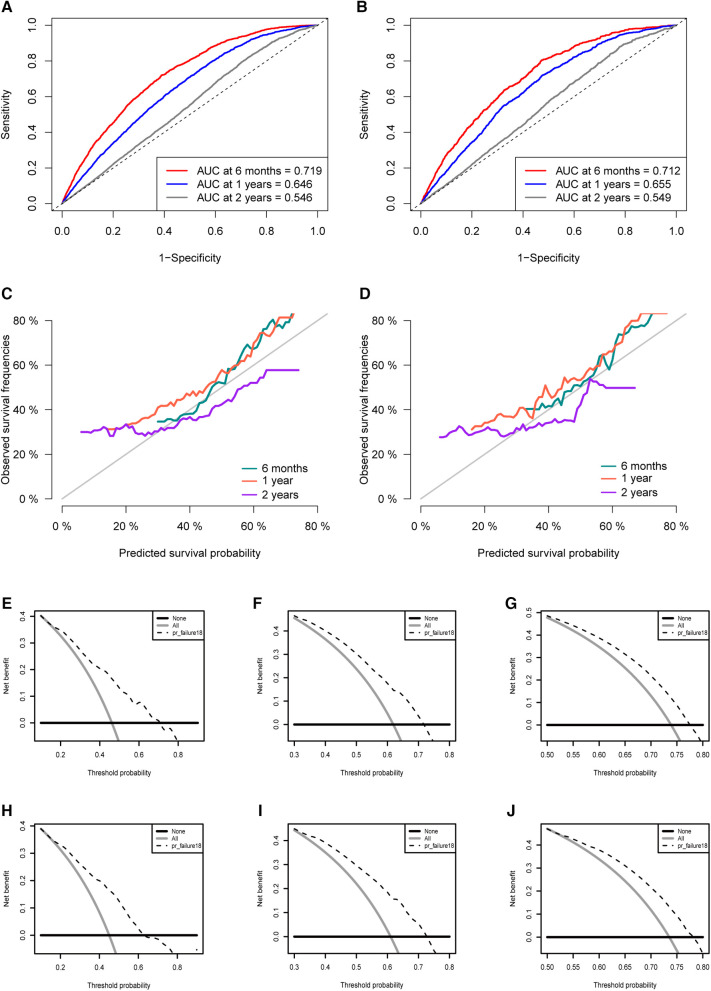
Area under the receiver operating characteristic curve for the training set (**A**) and validation set (**B**). Calibration curves in the training set (**C**) and validation set (**D**). Decision curves for half a year (**E**), 1 year (**F**), 2 years (**G**) in the training set. Decision curves for half a year (**H**), 1 year (**I**), 2 years (**J**) in the validation set.

To make the model more practical in clinical practice, we developed a nomogram of competing risk models. In our nomogram, there are eight clinical variables, including age, the specific site of spPDAC occurrence, interval, T stage, M stage, surgery, chemotherapy, and radiotherapy (Figure 6). The probability of occurrence of PDACSD in 6 months, 1 year, and 2 years can be predicted only by adding the scores of each variable of spPDAC patients. We used the C-index to verify the accuracy of the model. The C-index values were 0.665 (95% CI, 0.655, 0.675) and 0.666 (95% CI, 0.650, 0.682) for the training and validation sets, respectively. This showed that the model has a better discriminative ability. The training set and validation set AUC showed that our model has good discrimination (Figures 7A,B). The calibration curves showed that the predicted and actual observed values of the model were almost consistent (Figures 7C,D). DCA (Figures 7E–J) showed that the model had good clinical utility in predicting 6-month, 1-year, and 2-year PDACSD.

## Discussion

In this study, we first analyzed the impact of previous cancer history on the prognosis of patients with PDAC. The results suggest that PDAC patients without a previous history of cancer have a better prognosis. The difference in prognosis between the spPDAC subgroup and the pPDAC subgroup also implies that previous studies on the prognostic characteristics of the pPDAC patient population were not applicable to the spPDAC population. Therefore, developing a prediction model suitable for the spPDAC population is of great significance for the precise treatment of spPDAC.

We used the Fine and Gray proportional subdistributed hazard method to identify risk factors significantly associated with PDACSD, including age, race, interval, location, T stage, M stage, chemotherapy, and radiotherapy. We constructed a competing risk model nomogram to assess the probability of developing PDACSD in spPDAC patients.

We identified the three most common FPCs in spPDAC patients followed by prostate cancer, breast cancer, and digestive malignancies. Similar to our conclusions, He et al. (25) found in a retrospective study that the most common sites of previous cancer in spPDAC patients were the prostate, breast, kidney, and bladder. Prostate cancer is the most common site, probably because of its higher incidence and better prognosis (26, 27). These key populations should be carefully screened.

Jo et al. (28) conducted a retrospective cohort study and found that the mean age of patients with spPDAC (*n* = 110) was significantly higher (66.5 vs. 62.2 years) compared with pPDAC patients (*n* = 1,606, *p* < 0.001). In our study, age was an important risk factor for developing PDACSD in spPDAC patients (*p* < 0.001). In all spPDAC samples, the mean age (SD) of patients was 72.67 (9.64) years old. In the training set, patients 65 years or older had a higher risk of developing PDACSD (sdHR = 1.225, 95% CI, 1.121–1.338). Studies have shown that there are significant differences in the treatment decisions and clinical prognosis of PDAC with different ages, and PDAC is age-dependent cancer (29). Age is considered an independent prognostic factor for PDAC (29, 30).

PDAC has always been a very malignant tumor. In the United States, PDAC is the third leading cause of cancer-related death (31). Due to the highly aggressive nature of PDAC, patients often have local invasion and distant metastasis at the time of diagnosis, resulting in poor prognosis in PDAC patients (32). According to research statistics, the average survival time after PDAC diagnosis is only 6–9 months (33–35). At the end of our 3-year follow-up, 94.26% of patients had died, including 82.68% of PDACSD. This also supports the characteristics of high malignancy and poor prognosis of PDAC.

The treatment methods of PDAC mainly include surgery, chemotherapy, radiation therapy, immunotherapy, and targeted therapy. Radical surgical resection is the most effective method for PDAC (36), but only 20% of patients achieve effective remission with surgical treatment (37). At present, there is no authoritative organization to formulate surgical treatment standards for spPDAC. Doctors often judge whether a patient can undergo surgical treatment according to the patient's physical condition and tumor progression, combined with the surgical treatment standards for PDAC(38–40). Therefore, for the special group of spPDAC, more research and authoritative diagnosis and treatment standards are urgently needed. Standard FOLFIRINOX or gemcitabine-based combination chemotherapy can slightly improve overall survival, but most patients die from disease progression (41–43). In recent years, preoperative neoadjuvant therapy for PDAC has gained wide acceptance (44–46). Studies have reported that preoperative neoadjuvant radiotherapy and chemotherapy can improve the resectability of locally advanced PDAC (47, 48). In this study, patients who underwent surgery, chemotherapy, and radiotherapy had a relatively lower probability of PDACSD, and their sdHR (95% CI) values were 0.518 (0.469–0.573), 0.733 (0.678–0.793), and 0.888 (0.810–0.973), respectively. Targeted therapy has developed rapidly in the treatment of breast and ovarian cancer, enabling treatment in a precise manner (49, 50). However, for PDAC, targeted therapy has been slow to develop, and the only approved precision therapy drug, erlotinib, has only marginally improved survival (51, 52). Not only that, but immunotherapy has a limited role in PDAC (53). Humans still have a long way to go in the treatment of PDAC.

The median time interval (IQR) between diagnosis of FPC and spPDAC was 55 (28, 89) months. To avoid the possibility of synchronous transfer, we only selected samples with time intervals greater than 6 months for study. The shortest and longest intervals were 7 months and 180 months, respectively. Our study found that the longer the interval (month), the higher the risk of developing PDACSD in spPDAC patients (*p* = 0.016).

Due to the lack of reliable criteria for evaluating spPDAC, clinicians often make empirical judgments based on imaging studies, TNM staging, and the patient's physical condition (54, 55). Through multivariate Cox regression analysis, He and his colleagues (25) identified age (*p* < 0.001), sex (*p* < 0.001), race (*p* < 0.001), tumor size (*p* < 0.001), prior history of cancer (*p* < 0.001), SEER stage (*p* < 0.001), grade (*p* < 0.001), surgery (*p* < 0.001), chemotherapy (*p* < 0.001), and radiotherapy (*p* < 0.001) were the risk factors affecting the overall survival of patients. In competing risk events, He et al. used traditional analytical methods (Cox regression analysis and Kaplan–Meier analysis), which tended to overestimate the probability of PDACSD, creating a competing risk bias. This kind of research bias is not uncommon, and one study found that this error may occur in 46% of the literature (22). Patients with spPDAC may die from other causes such as traffic accidents and cardiovascular disease. For these causes of death, spPDAC did not contribute, and these causes of death could not be combined with PDACSD to analyze risk factors for spPDAC. Therefore, in competing risk events, the Fine and Gray proportional sub-distributed hazard method is advocated (56, 57). To the best of our knowledge, researchers have used the Fine and Gray proportional subdistributed hazard method to construct competing risk models for multiple primary cancers associated with cervical cancer (58) and colorectal cancer (59). However, competing risk models for spPDAC cancer-specific mortality have not yet emerged. Hopefully, our results can fill this gap.

Although we investigated risk factors associated with PDACSD and established a good prognostic prediction model for spPDAC, there are inevitably some deficiencies. First, this is a retrospective study, and some selection differences cannot be avoided, which may lead to specific biases. Second, the SEER database lacks some key information related to PDACSD, such as smoking, alcohol consumption, obesity, type II diabetes, tumor markers, surgical methods, chemoradiotherapy regimens, immunotherapy, and so on. (60–62). This prevents us from analyzing patient information comprehensively. Finally, our model needs to be validated in a large-scale prospective study.

## Conclusions

In conclusion, we analyzed the impact of previous cancer on the prognosis of spPDAC, screened risk factors for PDACSD in spPDAC patients, and constructed a competing risk model. The model has good accuracy and discriminative ability, which can assist doctors and patients in clinical decision-making.

## Data Availability

Publicly available datasets were analyzed in this study. This data can be found here: All data used in this work can be acquired from the SEER database (SEER: https://seer.cancer.gov/). To download SEER*Stat, visit http://seer.cancer.gov/seerstat/download. The author Lishan Song has gotten access to the SEER database (accession number:23514-Nov2020). Select the following datasets: Incidence - SEER Research Plus Data, 18 Registries (excl AK), Nov 2020 Sub (2000–2018)—Linked To County Attributes—Total US, 1969–2019 Counties, National Cancer Institute, DCCPS, Surveillance Research Program, released April 2021, based on the November 2020 submission.
